# A statistics-based reconstruction of high-resolution global terrestrial climate for the last 800,000 years

**DOI:** 10.1038/s41597-021-01009-3

**Published:** 2021-08-27

**Authors:** Mario Krapp, Robert M. Beyer, Stephen L. Edmundson, Paul J. Valdes, Andrea Manica

**Affiliations:** 1 Department of Zoology, University of Cambridge, Downing Street, Cambridge, CB2 3EJ United Kingdom; 2 GNS Science, PO Box 31312, Lower Hutt, 5040 New Zealand; 3 Department of Earth Sciences, Utrecht University, Budapestlaan 4, 3584 CD Utrecht, The Netherlands; 4 School of Geographical Sciences, University of Bristol, BS8 1SS Bristol, United Kingdom

**Keywords:** Palaeoclimate, Climate-change ecology, Climate and Earth system modelling

## Abstract

Curated global climate data have been generated from climate model outputs for the last 120,000 years, whereas reconstructions going back even further have been lacking due to the high computational cost of climate simulations. Here, we present a statistically-derived global terrestrial climate dataset for every 1,000 years of the last 800,000 years. It is based on a set of linear regressions between 72 existing HadCM3 climate simulations of the last 120,000 years and external forcings consisting of CO_2_, orbital parameters, and land type. The estimated climatologies were interpolated to 0.5° resolution and bias-corrected using present-day climate. The data compare well with the original HadCM3 simulations and with long-term proxy records. Our dataset includes monthly temperature, precipitation, cloud cover, and 17 bioclimatic variables. In addition, we derived net primary productivity and global biome distributions using the BIOME4 vegetation model. The data are a relevant source for different research areas, such as archaeology or ecology, to study the long-term effect of glacial-interglacial climate cycles for periods beyond the last 120,000 years.

## Abstract (summary)

**Table:** 

Measurement(s)	temperature • precipitation • cloud cover • net primary productivity
Technology Type(s)	computational modeling technique
Factor Type(s)	atmospheric carbon dioxide • orbital parameters • land surface type (ocean, land, land ice)
Sample Characteristic - Environment	climate system
Sample Characteristic - Location	Earth (planet)

Machine-accessible metadata file describing the reported data: 10.6084/m9.figshare.15073479

## Background & Summary

Studying the ecology and environment throughout past climatic changes often involves environmental reconstructions that are either based on paleoclimate proxies or on paleoclimate simulations. Unfortunately, even by today’s standards, simulating the climate over periods of thousands or hundreds of thousands of years in a continuous way can still be a costly and time-consuming endeavour. Present or future climate simulations are based on comprehensive Global Climate Models (GCMs) that resolve processes at high temporal and spatial resolution, such as those used in the fifth IPCC Assessment Report^1^. Climate model reconstructions for longer, continuous periods back in time are, therefore, challenging. They have to span a much longer period and are, thus, computationally too expensive. Instead, GCMs provide snapshots for a specific time or short transients in the order of a few thousand years. For longer, transient simulations of tens or hundreds of thousands of years, we rely on simulations from Earth System Models of Intermediate Complexity (EMICs)^2,3^ but they come at the cost of lower spatial resolution and a simplified representation of the climate system^4^.

Although there are a some high-resolution paleoclimate data sets readily available for download, for example, *WorldClim*^5^, *PaleoClim*^6^, or *ecoClimate*^7^, their temporal coverage is limited to a few snapshots of key periods in the past, for example, Mid-Holocene (6,000 years before present (BP)) the Last Glacial Maximum (21,000 years BP), or the Last Interglacial Period (130,000 years BP). An exception is *PaleoView*^8^ which covers the transient period of the last deglaciation, but this only goes back 21,000 years. Longer, continuous climate data sets of the past, based on HadCM3^9^ snapshots, have become available more recently, for example a Northern Hemisphere data set for the last 60,000 years^10^ or a bias-corrected, high-resolution terrestrial climate data set of the last 120,000 years^11^.

Here, we used a linear regression model to extend existing HadCM3 climate simulations of the last 120,000 years to create climate reconstructions of the last 800,000 years. We then applied a bias correction^12^ of the model output using present-day gridded observational data (CRU TS v. 4.04^13^) to downscale climate output to a final horizontal resolution of 0.5°^11,13^. This new data set is complementary to the aforementioned high-resolution terrestrial climate data set of the last 120,000 years^11^ (which should be preferred for studies of the last glacial cycle, as they are based directly on the GCM output), and it is an extension for readers to explore the climate history for earlier periods of the past.

In this paper, we present annual and monthly mean climatologies for the last 800,000 years in 1,000 year time steps (Table 1). The data set includes air temperature, precipitation, total cloud cover, 17 bioclimatic variables^14^, as well as biomes and annual and monthly net primary productivity, the latter based on BIOME4 simulations^15^, run on the debiased climatologies. We validated the long-term climate change signal using time series of various proxy records.

**Table 1:** Available reconstructions of environmental variables.

Variable	Unit
**Dimensional variables**	
longitude (720)	degrees east
latitude (360)	degrees north
time (800)	years before present
**Climatic variables**	
monthly temperature (Jan-Dec)	K
monthly precipitation (Jan-Dec)	mm year^−1^
monthly cloudiness (Jan-Dec)	%
minimum annual temperature	K
**Vegetation variables**	
monthly net primary productivity	gC m^−2^ month^−1^
annual net primary productivity	gC m^−2^ year^−1^
biome	categorical
**Bioclimatic variables**	
BIO1: annual mean temperature	°C
BIO4: temperature seasonality	°C
BIO5: minimum annual temperature	°C
BIO6: maximum annual temperature	°C
BIO7: temperature annual range	°C
BIO8: mean temperature of the wettest quarter	°C
BIO9: mean temperature of driest quarter	°C
BIO10: mean temperature of warmest quarter	°C
BIO11: mean temperature of coldest quarter	°C
BIO12: annual precipitation	mm year^−1^
BIO13: precipitation of wettest month	mm year^−1^
BIO14: precipitation of driest month	mm year^−1^
BIO15: precipitation seasonality	—
BIO16: precipitation of wettest quarter	mm year^−1^
BIO17: precipitation of driest quarter	mm year^−1^
BIO18: precipitation of warmest quarter	mm year^−1^
BIO19: precipitation of coldest quarter	mm year^−1^
**Land/land ice/ocean mask**	
mask	categorical

All variables have the dimensions 720 × 360 × 800 (longitude, latitude, time). Temperature seasonality (BIO4) and precipitation seasonality (BIO15) are given by the standard deviation of monthly temperatures and by the coefficient of variation of monthly precipitation, respectively. Temperature annual range (BIO7) is given by the difference between maximum annual temperature (BIO5) and minimum annual temperature (BIO6). Unit abbreviations: mm (millimetres), m (metres), gC (grams carbon).

## Methods

Our climate reconstructions are based on a set of linear regression models for each of the HadCM3 model grid boxes (N = nlon × nlat = 96 × 73 = 7008). Each linear model predicts a climate variable, which can be either temperature, precipitation, or total cloud cover, i.e., the dependent variable. The independent variables, i.e., the forcing terms, of the model are three orbital parameters, atmospheric CO_2_, and a surface type mask (land, ocean, or land ice), five variables in total.

Each linear model uses 72 data points given by the HadCM3 snapshots throughout the past 120 thousand years (ka)^16,17^. These snapshots cover both the Last Glacial Maximum, one of the coldest glacial stages, and the Last Interglacial, one of the warmest interglacial stages during the Middle and Late Pleistocene. By applying available long-term forcing to the solutions of the linear models, we reconstructed the climate for periods before 120 ka. The forcing consists of CO_2_^18^, interpolated to 1 ka intervals for the last 800 ka, orbital parameters, taken from numerical solution to the Earth’s orbit around the sun^19^, and surface type masks based on numerical ice-sheet model output^2^ and a global sea-level record^20^. At this stage, the reconstructed climate of the last 800,000 years has the same coarse spatial resolution as the underlying HadCM3 snapshots. In a last step, we applied a bias correction (including spatial downscaling) for the terrestrial climate to derive a spatially explicit data set that covers the last 800 ka in 1 ka intervals with a spatial resolution of 0.5° × 0.5°. For each variable, these steps were repeated for each monthly mean climatology (Jan–Dec), as well as for the annual mean values (Table 1).

**Fig. 1:**
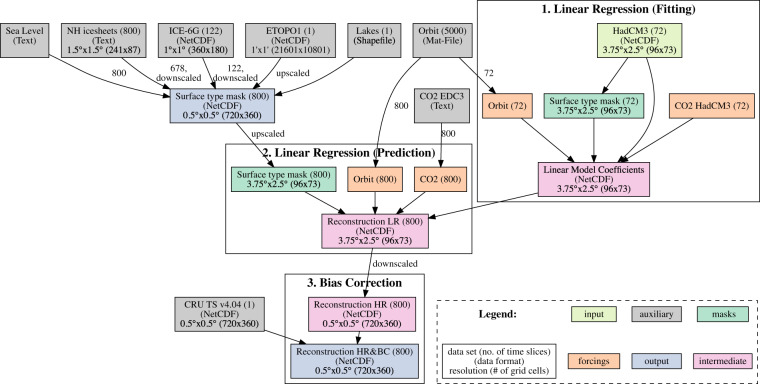
Flowchart showing how the different data sets have been used as input for the different stages of the paleo-climate data generation: A linear regression combines 72 low-resolution (LR) HadCM3 snapshot simulations with the external forcings, i.e., CO_2_, orbital parameters, and surface type masks (ocean, land, land ice), which provides the basis of the long-term climate reconstructions using long-term forcings and surface type masks. The final bias correction procedure yields the high-resolution (HR), bias-corrected (BC) climate data set for the last 800 ka.

A comprehensive overview of our approach is shown in Fig. 1 and further details of our experimental setup are given below.

### The HadCM3 climate model

HadCM3 is a fully coupled global climate model with an atmospheric component, HadAM3, which has a horizontal resolution of 3.75° × 2.5°, 19 vertical levels, and a time step of 30 minutes. The ocean and sea-ice component of HadCM3 has a horizontal resolution of 1.25° × 1.25° and 20 vertical levels. HadCM3 simulations were run with a prescribed ice-sheet and continental geometry. We used output from the atmospheric component of the 72 available HadCM3 simulations covering the last 120,000 years in 2,000-year intervals from 120,000 to 24,000 years BP and in 1,000-year intervals from 22,000 years BP to present-day^16,17^.

### Surface type mask: Ice-sheet extents, sea level and lakes

As ice sheet extents for the period outside the HadCM3 snapshots, we used model outputs from CLIMBER-2/SICOPOLIS simulations^2^ for which Northern Hemisphere ice sheet extents and heights are available for the last 800 ka in 1 ka-year intervals. For the more recent period from 122–0 ka, we used the ice sheet configurations from the ICE-6G data set^21^ (http://www.atmosp.physics.utoronto.ca/ peltier/data.php). Changes in the coast lines affecting the land–sea mask were derived from a global sea-level record^20^. We overlaid those changes on top of present-day coast lines, taken from the ETOPO1 data set^22^ (https://ngdc.noaa.gov/mgg/global/global.html), while we preserved inland lakes which were taken from the *Global Lakes and Wetlands Database*^23^ (https://www.worldwildlife.org/pages/global-lakes-and-wetlands-database).

### Training and test data

**Fig. 2:**
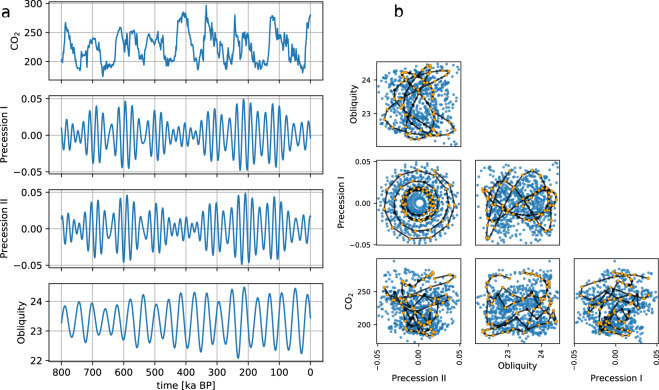
(**a**) Time series of the four external parameters: CO_2_ and orbital parameters for the last 800 ka and (**b**) the associated parameter space as scatter plot matrix (blue dots). The continuous CO_2_ record is from the EPICA Dome C ice core in Antarctica^18^. The orbital parameters are numerical solutions for the Earth’s orbit and rotation in terms of eccentricity, precession, and obliquity^19^. In (**b**), black lines with black dots represent the total 72 parameter sets. Orange dots highlight the parameter sets of the 58 HadCM3 snapshot simulations which we used as training data (80% of the total 72) for the linear regression model.

We divided the HadCM3 snapshots into a training (80%) and a test data set (20%). The training data set was used to fit the linear model while the test data was used for a comparison to the reconstructed snapshots. For a 80/20 division of the 72 time slices into training and test data, i.e., 58/14, there are $$\left(\begin{array}{c}n\\ k\end{array}\right)=\left(\begin{array}{c}72\\ 58\end{array}\right)\approx 3\times 1{0}^{14}$$ possible combinations of snapshots. But instead of randomly dividing the snapshots into the training/test data, we followed an approach with the aim to preserve as much variance as possible in the training data, i.e. maximise the variance of the predictors. This is best illustrated by the phase plots of the parameters, i.e., the predictors (Fig. 2). The training data set covers the edges of each phase plane and thus maximises the phase space covered by the linear regression model. This choice of training data ensured that the linear regression model interpolated within the phase space and did not need to extrapolate for the test data.

The procedure was as follows. We calculated the covariance matrix of the full parameter set (*n* = 72), *C*_*full*_. Then, we randomly created a sample training data set (*k* = 58) for which we computed the covariance matrix *C*_*sample*_. If the eigenvalues of *C*_*sample*_ were larger than the eigenvalues of *C*_*full*_, then the training sample data set contained at least as much variance as the full data set and this sample training data set was marked as a candidate for the final training set. After several iterations (*N* = 10,000), we summed up how many times each time slice had appeared within a candidate training set. We then ranked all time slices according to this number. In the final step, we picked the 80% top-ranked time slices as training data.

Note, the division into training and test data set was made only for the validation of the linear model. For the actual climate reconstructions with the linear regression model, we used all 72 snapshots to make full use of the complete set of available data.

### The linear regression model

For each HadCM3 grid box, we fitted a linear regression model to a local series of each climatic variable of interest (temperature, precipitation, or cloud cover) with the following independent variables or forcings: atmospheric CO_2_ concentrations (as a major greenhouse gas), three variables reflecting the orbital forcing^19^, and the surface type, which is either ocean, land, or land ice. The orbital parameters are obliquity *ε* and two combinations of eccentricity *e* and precession *ω*: *e* · sin *ω*, henceforth referred to as precession index I, and *e* · cos *ω* (precession index II), and they are a generally accepted set of orbital forcings^24,25^. We chose temperature *T*, precipitation *P*, and total cloud cover *C* as dependent variables. The independent variables are given by the normalised forcings.

More formally, let *Y*(*x, t*) be a time series of a climate variable in a specific grid box *x* at time *t*. Our linear model should explain variations of *Y*(*x, t*), Δ*Y*(*x, t*), around a mean value $\bar{Y(x,t)}$:

1 $$\Delta Y(x,t)=Y(x,t)-\bar{Y(x,t)}.$$

To make the linear model well-conditioned, all independent variables were normalised. The mean was subtracted and the result were then divided by the standard deviation.

Precipitation and total cloud cover are bounded variables which can lead to linear model predictions outside of physically meaningful ranges. A common procedure to prevent these out of bounds predictions is to apply a transformation to the data beforehand. To prevent the linear model from predicting negative precipitation values, we therefore applied a logarithmic transformation to precipitation, which maps values from [0, +∞] to [−∞, +∞]. Thus, in the case of precipitation, the linear regression coefficients predict the response in terms of anomalies in the exponent. For total cloud cover, expressed as fraction between 0 and 1, we choose a logit transformation which maps values from [0, 1] to [−∞, +∞]. Note that the minimum values for precipitation and total cloud cover, as simulated by HadCM3, are never exactly zero (or smaller), except at the poles, 90° N/S, which were excluded for that reason; therefore, the transformations always yield finite values. The decomposition of temperature *T*, precipitation *P*, and total cloud cover *C*, i.e., the Δ*Y*(*x, t*) on the left hand side of Eq. (1) is:

2 $$T(x,t)=\bar{T(x,t)}+\mathop{\underbrace{\Delta T(x,t)}}\limits_{\hat{=}\Delta Y(x,t)}$$

3 $$\log (P(x,t))=\bar{\log (P(x,t))}+\mathop{\underbrace{\Delta \log (P(x,t))}}\limits_{\hat{=}\Delta Y(x,t)}$$

4 $${\rm{\log }}\left(\frac{C(x,t)}{1-C(x,t)}\right)=\overline{{\rm{\log }}\left(\frac{C(x,t)}{1-C(x,t)}\right)}+\mathop{\underbrace{\Delta {\rm{\log }}\left(\frac{C(x,t)}{1-C(x,t)}\right)}}\limits_{\widehat{=}\Delta Y(x,t)}$$

The linear regression model for each (transformed) anomaly is:

5 $$\begin{array}{lll}\Delta Y(x,t) & = & \mathop{\underbrace{{\beta }_{1}(x)\cdot \Delta \varepsilon (t)}}\limits_{{\rm{obliquity}}\;{\rm{forcing}}}+\mathop{\underbrace{{\beta }_{2}(x)\cdot \Delta (e\cdot {\rm{\sin }}\,\omega )(t)}}\limits_{{\rm{precession}}\;{\rm{index}}\;{\rm{I}}\;{\rm{forcing}}}+\mathop{\underbrace{{\beta }_{3}(x)\cdot \Delta (e\cdot {\rm{\cos }}\,\omega )(t)}}\limits_{{\rm{precession}}\;{\rm{index}}\;{\rm{II}}\;{\rm{forcing}}}\\  &  & +\mathop{\underbrace{{\beta }_{4}(x)\cdot \Delta C{O}_{2}(t)}}\limits_{{\rm{greenhouse}}\;{\rm{gas}}\;{\rm{forcing}}}+\mathop{\underbrace{{\beta }_{5}(x)\cdot M(x,t)}}\limits_{{\rm{surface}}\;{\rm{type}}}\end{array}$$

**Fig. 3:**
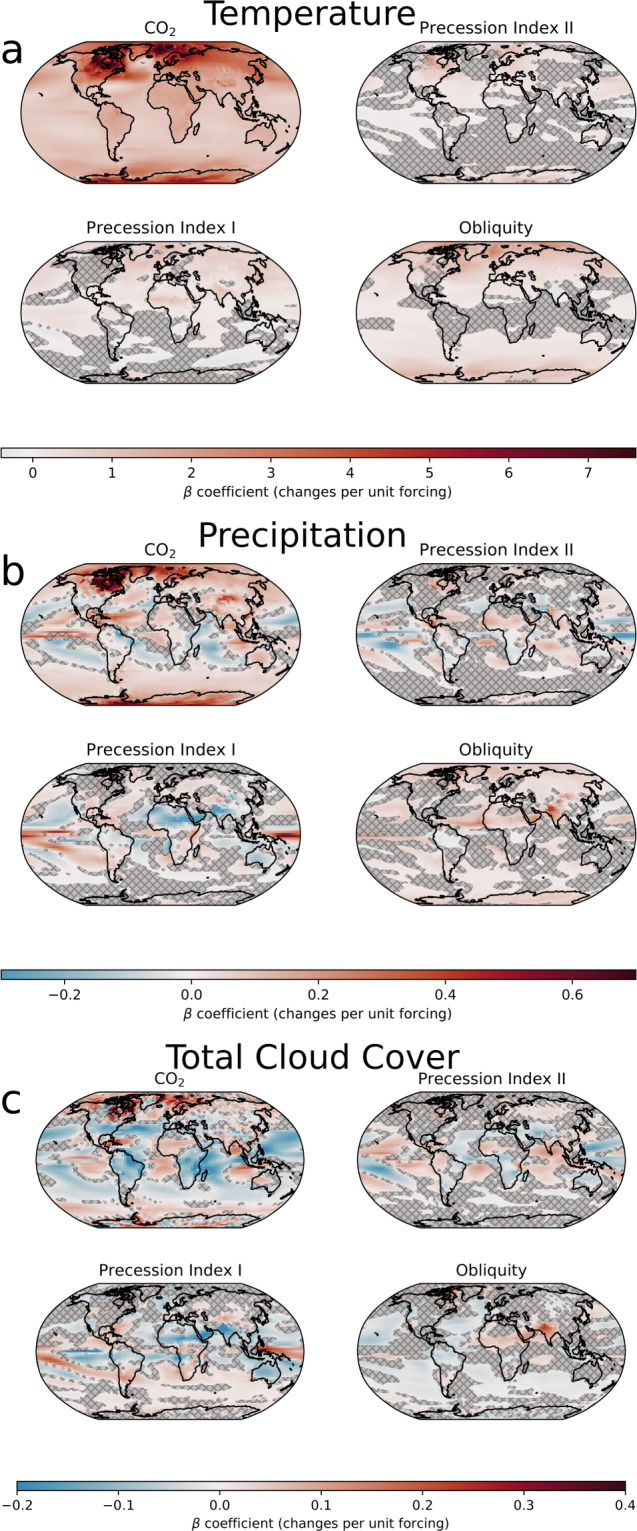
Regression coefficients, i.e., *β* coefficients, for (**a**) mean annual temperature, (**b**) precipitation, and (**c**) total cloud cover. Regions where the respective coefficient is not statistically significant (p < 0.05) are hatched and shaded.

Here, *β*_1_ to *β*_5_ are the regression coefficients for the respective predictors (see Fig. 3 for maps of *β* coefficients). Surface type changes are captured by the categorical variable *M*(*x, t*) ∈ [ocean, land, land ice]. For example, around coastlines land grid boxes can turn into ocean grid boxes when sea level is high. Similarly, expanding ice sheets turn land grid boxes into ice-covered grid boxes, and the climate variable *Y*(*x, t*) may respond to different surface types in different ways. The categorical variables were encoded using *Treatment* coding. The first level, ocean, was chosen as a reference level and is by definition zero *β*_5_(*x*) = 0. For the other two levels, land and land ice, a different *β*_5_(*x*) was assigned for each—in effect, a different intercept for Δ*Y*(*x, t*).

We solved the linear model for each transformed variable, applied the extended forcing to generate the 800 ka climate reconstructions, and transformed the resulting data back to its original range according to Eqs. (2–4). At this stage, we have an extended HadCM3 model output of annual and monthly mean for temperature, precipitation, and total cloud cover, still at the original HadCM3 resolution, for the last 800 ka.

### Spatial downscaling to 0.5° and bias correction

#### The CRU TS climate data set

For the bias correction of the extended HadCM3 model output, as predicted by the previously described linear model, we used variables from the CRU TS (Climatic Research Unit Timeseries) data set (v. 4.04)0^13^. Before the bias correction step, the data set has been bi-linearly interpolated from the spatial resolution of 3.75° × 2.5° to the CRU TS resolution of 0.5° × 0.5°. CRU TS v. 4.04 contains monthly time series fields of precipitation, daily maximum and minimum temperatures, cloud cover, and other variables covering all land areas (except Antarctica) for 1901 to 2019. As reference period for the bias correction we chose 1961–1990. We applied the additive “delta” method, which is the most effective bias correction with respect to paleoclimate reconstructions^12^, to all climate variables predicted by the linear model (subscript LM) to create the final, bias-corrected climate data set $\widehat{Y}(x,t)$:

6 $$\widehat{Y}(x,t)=Y{(x,t)}_{{\rm{LM}}}+\left[Y{(x,0)}_{{\rm{CRU}}{\rm{TS}}}-Y{(x,0)}_{{\rm{LM}}}\right].$$

#### The BIOME4 vegetation model

We used the BIOME4 model^15^ to calculate annual net primary productivity (NPP) and to determine the global distribution of biomes. BIOME4 is a coupled biogeography and biogeochemistry model that simulates competition of different plant functional types (PFTs). It optimises the leaf area of each PFT as a function of NPP. BIOME4 forcing consists of monthly values of temperature, precipitation, and sunshine percentage, as well as values of annual minimum temperature and atmospheric CO_2_ concentrations. Sunshine percentage was computed using total cloud cover^26^. For atmospheric CO_2_ we used the same data set as described earlier^18^. In its default setup, BIOME4 does not incorporate orbital variations that would affect top-of-atmosphere (TOA) insolation. Instead, it is approximated by a cosine function representative of present-day insolation only. We therefore updated the TOA insolation representation in BIOME4 so that it takes changes in the Earth’s orbit into account^27^. Further inputs, which were kept constant through time, are water holding capacity and percolation rate.

## Data Records

All data records are publicly available as NetCDF files in the project repository in the data directory^28^.

### Monthly mean and annual mean climatologies

The climate variables that are part of our data set are listed in Table 1 and can be downloaded as NetCDF files (Table 2) from the *Open Science Framework* data repository^28^. All climate variables are available on a 0.5° × 0.5° resolution, i.e., the same regular grid as the CRU TS v4.04 data set^13^.

**Table 2:** List of data sets that can be found in the *Open Science Framework* repository [28] under the project’s data directory.

**Reconstructed and bias-corrected 800 ka outputs**
temp_800ka_jan.nc
…
temp_800ka_dec.nc
temp_800ka_min.nc
temp_800ka_ann.nc
prec_800ka_jan.nc
…
prec_800ka_dec.nc
prec_800ka_ann.nc
tcc_800ka_jan.nc
…
tcc_800ka_dec.nc
tcc_800ka_ann.nc
bio01_800ka.nc
bio04_800ka.nc
bio05_800ka.nc
…
bio19_800ka.nc
**BIOME4 800 ka outputs**
biome4output_800ka.nc
biome4output_800ka_jan.nc
…
biome4output_800ka_dec.nc
**Land/land Ice/ocean masks**
icesheets_000-800_cru.nc

### Bioclimatic variables

For ecological applications such as species distribution modelling, bioclimatic variables are more relevant than the actual climate variables because they capture information about annual and seasonal climate conditions as reflected by temperature and precipitation^14^, for example coldest/warmest or wettest/driest quarter averages of precipitation and temperature. Most of the commonly required bioclimatic variables can be directly derived from monthly mean temperature and precipitation data, and are thus included in our data records (Tables 1, 2). *Annual Mean Diurnal Range* (BIO2) and *Isothermality* (BIO3) cannot be calculated from the available climate model data (HadCM3) and are therefore not included. For BIO2, we do not have the monthly minimum and maximum temperature, and BIO3 depends on BIO2 (BIO3 = BIO2/BIO7 × 100).

### Net primary productivity and biomes

Our data record contains annual and monthly net primary productivity as well as categorical biome data, both of which were calculated with the BIOME4 vegetation model, using the 800 ka of reconstructed and bias-corrected climate (Tables 1, 2).

## Technical Validation

### Comparison to original HadCM3 simulations

**Fig. 4:**
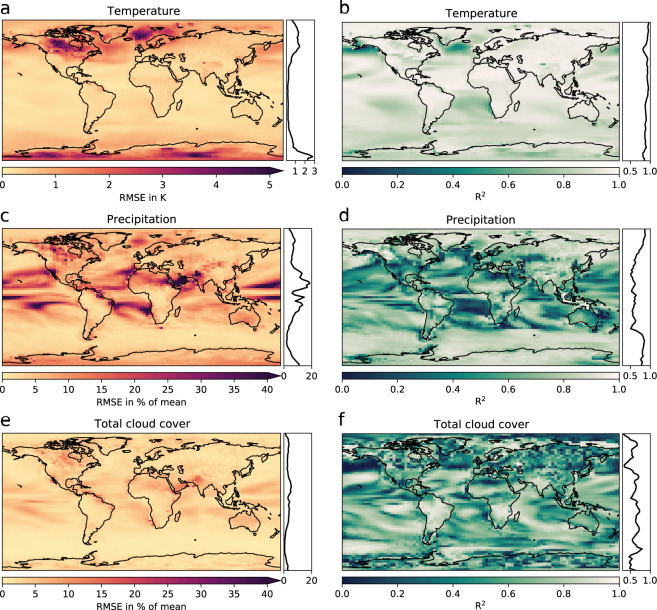
Left panel (**a**,**c**,**e**): Root mean square errors (RMSE) as estimators of the goodness of fit (lower is better) calculated using the test data. Right panel (**b,d,f **): *R*^2^ values as estimator for the goodness of the model (higher is better) using the training data. Shown are the *R*^2^ and RMSEs for (**a,b**) mean annual temperature, (**c,d**) mean annual precipitation and (**e,f**) mean annual total cloud cover. Note, that only the values over land and land ice areas are relevant for the overall quality of the final data product.

We validated our reconstruction from the linear model against the original HadCM3 snapshots. As comparison metric, we used *R*^2^ values, a goodness of fit estimator that measures the proportion of variance explained by the linear model, and the root mean squared error (RMSE), an estimator of the goodness of the model that measures how far the linear model predictions are from the HadCM3 test data (Fig. 4).

Overall, our linear model is a better predictor for temperature than for precipitation and total cloud cover. Temperature responds more directly to local forcings than precipitation and cloud cover, because it is determined by the energy balance of downward and upward longwave and shortwave radiation and turbulent heat fluxes. The downward shortwave radiation depends on incoming solar radiation that is determined by orbital variations, whereas downward longwave radiation is determined by greenhouse gases such as CO_2_ and water vapour, as well as cloud cover. Large-scale atmospheric circulation changes have a much smaller effect on temperature. The high *R*^2^ and low RMSE values in most regions (Fig. 4a,b) mean that temperature is locally well constrained by global CO_2_ and orbital variations and our linear model captures this effect well.

The matter is more complicated for precipitation and cloud cover. Both variables are directly affected by the hydrological cycle which itself depends on large-scale atmospheric dynamics, such as monsoonal systems in the tropics and subtropics, or mid-latitude storm systems. Local interactions between the atmosphere and the surface, such as evaporation and transpiration over the ocean, or deep convection over the tropics, matter to a lesser extent. Instead, processes and circulation features like moisture transport or the atmospheric Hadley cell dynamics determine the non-local response of precipitation (and cloud cover) to CO_2_ or orbital variations to a much larger extent. Because of the larger dynamical component of the hydrological cycle, precipitation and cloud cover are much less constrained by the forcing than temperature. As a result, the linear model shows less predictive skill for precipitation and total cloud cover (Fig. 4c–f).

Our reconstruction represented only long-term climatologies of past climate changes, similar to other GCM snapshot of the past^11,16^. Therefore, the data set does not contain sub-millennial scale variability.

### The spatial and temporal covariance of the model output

Our climate reconstructions are based on a pixel-specific linear model, one for each of the HadCM3 grid boxes. By design, spatial autocorrelation is not an issue, which it would be if we were to analyse all points simultaneously. In that case, spatial autocorrelation would invalidate the linear model as the residuals would be spatially autocorrelated.

**Fig. 5:**
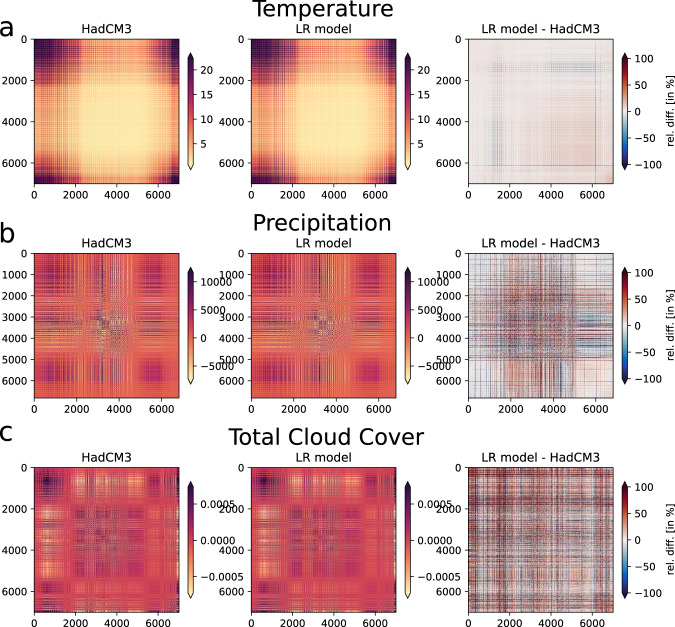
Spatial covariance matrix of (**a**) temperature (in units K^2^), (**b**) precipitation (in units mm^2^/a^2^), and (**c**) total cloud cover (in units of 1^2^) for HadCM3, our linear regression model (LR model), and the difference between the two. Each value represents a covariance matrix element from a flattened vector with the length of the total number of grid points (n = 7008). The covariance matrices are symmetric and thus are their differences.

However, HadCM3 exhibits a certain spatial structure, and such a pixel-by-pixel approach, where spatial grid cells are treated independently, cannot guarantee that this spatial structure, or covariance, is preserved. We can nevertheless show that the reconstructed climate fields exhibit the same spatial structure as the original HadCM3 model output. First of all, the regression coefficient maps (Fig. 3) indicate that the climate response to external forcings is spatially coherent. We calculated this spatial coherence in terms of the spatial covariance matrix (Fig. 5). It consists of the covariance between the time series of any two grid points, i.e., it is a 7008 × 7008 matrix (= 96 × 73 (nlon × nlat) = 7008) of covariances over the 72 snapshots.

For both HadCM3 and the linear model, the covariance matrices are similar in structure and magnitude, and their differences are relatively small (Fig. 5). The covariance matrices are much more similar for temperature than for precipitation and total cloud cover, because the linear regression works better for temperature than for the other two climate fields. Overall, the covariance matrices of the linear model reconstructions are so similar to HadCM3 that we can conclude that the spatial structure is indeed preserved.

**Fig. 6:**
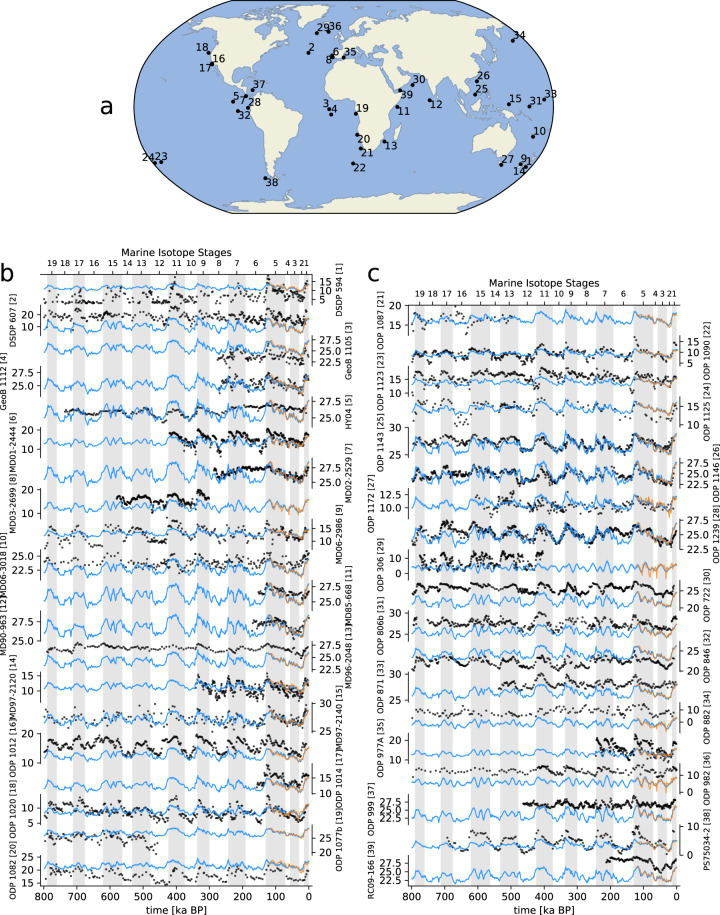
(**a**) Map of the 39 Middle and Late Pleistocene marine sea surface temperature proxies used in this study and their respective time series (**b,c**). Black dots indicate proxy sea surface temperature while blue lines indicate mean annual temperature as reconstructed for every 1 ka of the last 800 ka. Proxy–derived and model temperature are on the same scale, in). Orange lines are original time series from HadCM3. Grey bars indicate glacial stages. The coefficients for the correlation between the reconstructed temperature (blues lines) and the proxy record (black dots) can be found in Table 3.

**Fig. 7:**
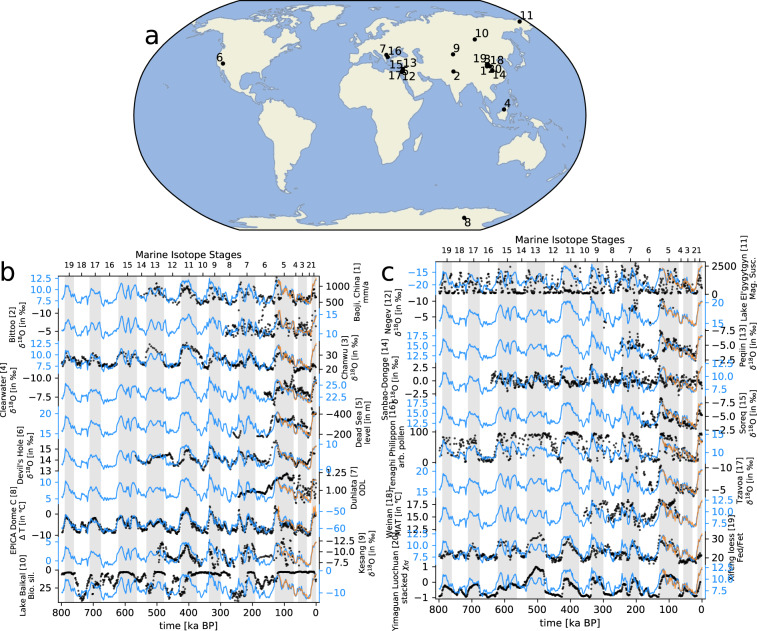
(**a**) Map of the 20 Middle and Late Pleistocene terrestrial climate proxies used in this study and their respective time series (**b**). Black dots indicate proxy variables (in different units) while blue lines indicate mean annual temperature as reconstructed for every 1 ka of the last 800 ka (in). Orange lines are original time series from HadCM3. Grey bars indicate glacial stages. The coefficients for the correlation between the reconstructed temperature (blues lines) and the proxy record (black dots) can be found in Table 4.

For any time series of observations (for example, as shown in Figs. 6, 7), we can assume that data points of that time series are temporally autocorrelated because of possible lag effects that derive from non-equilibrium climate dynamics. However, our reconstructions are based on snapshot simulations that are assumed to be in equilibrium, and any such lags are therefore omitted. The relationship between outputs of snapshots and their forcings can thus be treated as independent data points with no temporal autocorrelation.

### Comparison to marine and terrestrial proxies

We compared the reconstructed climate data with marine proxies (before the downscaling/bias-correction step) as a means of highlighting how well the reconstructed long-term climatologies compare to empirical reconstructions.

Marine sediment cores are valuable archives of past sea surface temperature (SST) records. Because their associated bio-geochemistry is relatively straightforward, marine proxies can be utilised as paleo-thermometers and are thus well suited for a direct proxy–model comparison. For these proxies, we compared model-derived mean annual temperature (MAT) time series directly with proxy-derived SST time series and calculated the correlation between the two. Note that MAT and SST are not the same climatological quantities; SST is the temperature of the ocean surface and has a lower limit of about −1.8°C, the freezing point of saltwater. While we expect MAT and SST to co-vary in low and mid-latitudes, at higher latitudes, seasonal or perennial sea ice cover makes a comparison between both variables problematic.

Although terrestrial proxies are rarely available as direct temperature estimates, we could still calculate the correlation between the model-derived and the proxy-derived time series. However, the interpretation of terrestrial proxies from a climate perspective can be problematic. For example, pollen-based vegetation reconstructions are suggested to be less reliable as climate proxies, particularly for interglacials^29^. Other land-based proxies such as dust deposits integrate long-term climatic changes over large regions and hence do not necessarily capture climatic effects at their specific location.

**Fig. 8:**
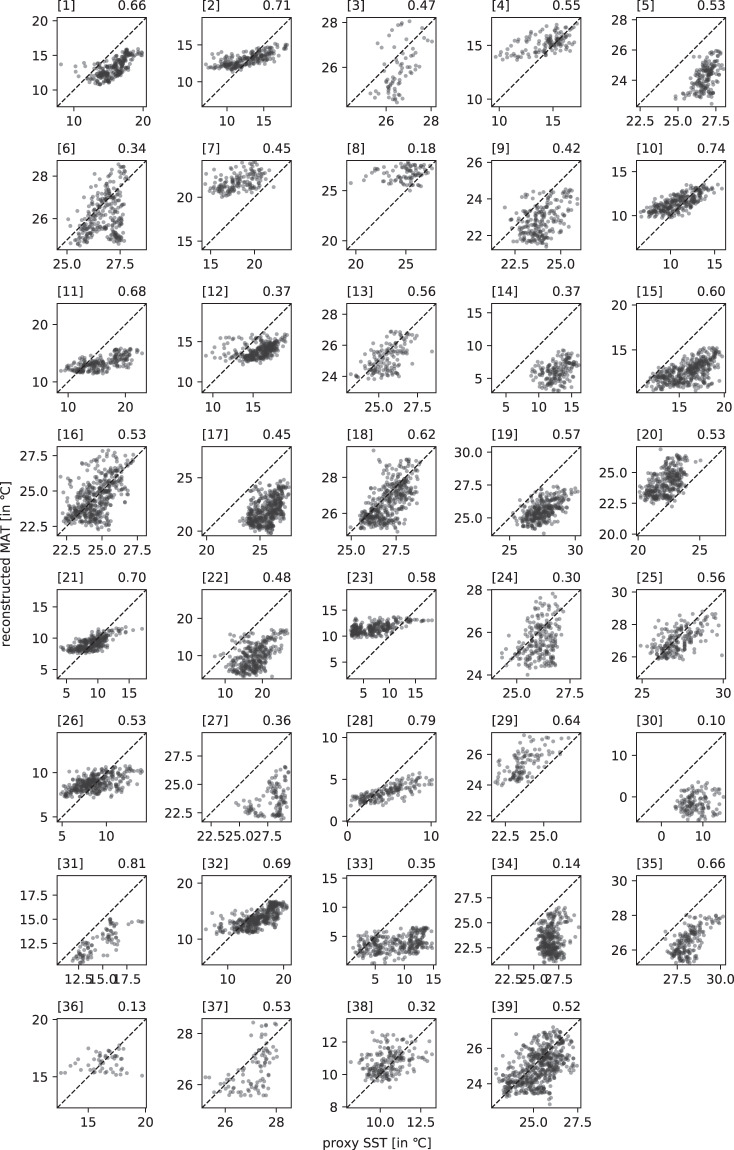
Scatter plot of the 39 Middle and Late Pleistocene marine proxies (x-axis) used in this study versus reconstructed mean annual temperature (y-axis). The respective correlation coefficient is shown on the top right in each plot and information about each proxy can be found in Table 3. The dashed diagonal line represents the hypothetical 1-1 for a perfect model.

**Fig. 9:**
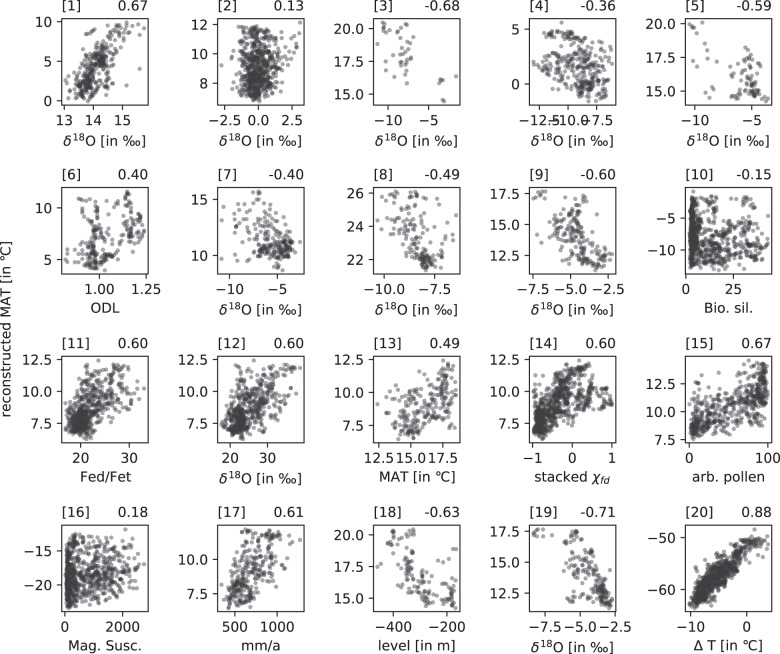
Scatter plot of the 20 Middle and Late Pleistocene terrestrial proxies (x-axis) used in this study versus reconstructed mean annual temperature (y-axis). The respective correlation coefficient is shown on the top right in each plot and information about each proxy can be found in Table 4.

For the comparison of our climate reconstructions with proxy reconstruction, we assembled long-term marine SST and terrestrial climate proxy reconstructions (Figs. 6, 7, 8, 9) that cover a period of at least 150 ka during the last 800 ka (Tables 3 and 4).

**Table 3:** Marine proxy records that have been used in the validation of the climate reconstruction, their coordinates, correlation coefficients, types, and respective references.

core/name	lon (°E)	lat (°N)	corr coeff	type	reference(s)
DSDP 594	175.0	−45.5	0.58	SST	^43,44^
DSDP 607	−33.0	41.0	0.48	SST	^43,45^
GeoB 1105	−12.4	−1.7	0.64	SST	^46^
GeoB 1112	−10.7	−5.8	0.56	SST	^46^
HY04	−95.0	4.0	0.30	SST	^43,47^
MD01-2444	−10.1	37.6	0.69	SST	^48^
MD02-2529	−84.1	8.2	0.34	SST	^49^
MD03-2699	−10.7	39.0	0.66	SST	^50^
MD06-2986	167.9	−43.4	0.71	SST	^43,51^
MD06-3018	166.2	−22.6	0.42	SST	^52^
MD85-668	46.0	0.0	0.47	SST	^53^
MD90-963	73.9	5.1	0.53	SST	^54^
MD96-2048	36.0	−26.2	0.53	SST	^55^
MD97-2120	174.9	−45.5	0.74	SST	^56^
MD97-2140	141.5	2.0	0.56	SST	^43,57^
ODP 1012	−118.4	32.3	0.60	SST	^43,58^
ODP 1014	−118.9	32.8	0.81	SST	^59^
ODP 1020	−126.4	41.0	0.53	SST	^43,60^
ODP 1077b	10.4	−5.2	0.18	SST	^61^
ODP 1082	11.8	−21.1	0.45	SST	^62^
ODP 1087	15.3	−31.5	0.13	SST	^63^
ODP 1090	8.9	−42.9	0.70	SST	^43,64^
ODP 1123	−171.5	−41.8	0.37	SST	^43,65^
ODP 1125	−178.2	−42.6	0.55	SST	^66^
ODP 1143	113.3	9.4	0.62	SST	^43,67^
ODP 1146	116.3	19.5	0.53	SST	^43,68^
ODP 1172	149.9	−44.0	0.32	SST	^69^
ODP 1239	−82.1	−0.7	0.52	SST	^70^
ODP 306	−27.9	56.4	0.35	SST	^71^
ODP 722	59.8	16.6	0.45	SST	^43,68^
ODP 806b	159.4	0.3	0.57	SST	^43,72^
ODP 846	−90.8	−3.1	0.53	SST	^43,73^
ODP 871	172.3	5.6	0.65	SST	^74^
ODP 882	167.6	50.4	0.10	SST	^75^
ODP 977 A	0.0	37.5	0.68	SST	^48^
ODP 982	−15.9	57.5	0.37	SST	^43,76^
ODP 999	−78.7	12.8	0.14	SST	^77^
PS75034-2	−80.1	−54.4	0.79	SST	^43,78^
RC09-166	48.8	12.5	0.36	SST	^79^

**Table 4:** Terrestrial proxy records that have been used in the validation of the climate reconstruction, their coordinates, correlation coefficients, types, and respective references.

core/name	lon (°E)	lat (°N)	corr coeff	type	reference(s)
Baoji, China	107.1	34.4	0.61	rainfall	^80^
Bittoo	77.8	30.8	−0.40	*δ*^18^O	^81^
Chanwu	107.7	35.2	0.60	*δ*^18^O	^82^
Clearwater	114.9	4.1	−0.49	*δ*^18^O	^83^
Dead Sea	35.0	30.5	−0.63	lake level	^84^
Devil’s Hole	−116.3	36.4	0.67	*δ*^18^O	^85^
Duhlata	23.2	42.5	0.40	ODL	^86^
EPICA Dome C	123.4	−75.0	0.88	temperature	^87^
Kesang	81.8	42.9	−0.36	*δ*^18^O	^88^
Lake Baikal	108.4	53.7	−0.15	Bio. sil.	^89^
Lake El’gygytgyn	172.0	67.5	0.18	mag. susc.	^90^
Negev	34.8	30.6	−0.68	*δ*^18^O	^91^
Peqiin	36.0	32.6	−0.60	*δ*^18^O	^92^
Sanbao-Dongge	110.4	31.7	0.12	*δ*^18^O	^93^
Soreq	36.0	31.4	−0.71	*δ*^18^O	^92^
Tenaghi Philippon	24.2	41.0	0.67	arb. pollen	^43,94^
Tzavoa	35.2	31.2	−0.59	*δ*^18^O	^95^
Weinan	109.6	34.4	0.49	temperature	^96^
Xifeng loess	107.6	35.7	0.60	Fed/Fet	^82^
Yimaguan Luochuan	108.5	35.8	0.60	mag. susc.	^43,97^

In locations where we have empirical proxies, both on land and over the ocean, our regression-based climate reconstructions match the original HadCM3 simulations well. These reconstructions can therefore be considered as representative of the simulated HadCM3 climate. As a consequence, any differences with respect to the proxies records will persist in our reconstructions and, therefore, needs to be removed.

### Model bias

**Fig. 10:**
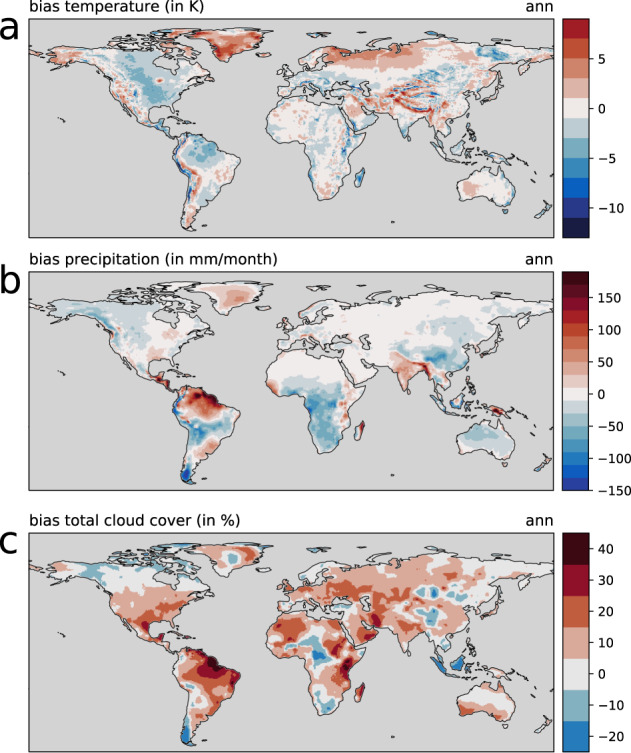
Model bias for (**a**) annual mean temperature, (**b**) annual mean precipitation, and (**c**) annual mean total cloud cover, as differences between the linear model reconstruction for 0 ka and present-day (average of 1961–1990) CRU TS data.

Because the linear model was fitted to match HadCM3 model outputs, the resulting bias of our reconstructions is similar to the HadCM3 model bias. The bias of reconstructed temperature and precipitation with respect to the CRU TS data set has a similar spatial pattern (Fig. 10) and is as large as the bias shown for HadCM3 climate reconstructions of the last 60 ka^10^. This is also why our long-term reconstructions show the same bias towards the assembled paleo-climate proxy records as the original HadCM3 simulations (Figs. 6, 7). From this, we concluded that this similarity means that our present-day climate reconstruction is of the same quality as the original HadCM3 simulation it is based on. As discussed earlier, the bias was removed from our climate reconstructions using the “delta” method^12^.

### Informed user notice

Our final dataset is just as good as i) the goodness of the applied linear regression model, and ii) the underlying climate model, HadCM3 in our case. How well the linear regression model performs for different variables and different regions can be seen in Fig. 4. It works usually better for temperature and thus for temperature derived bioclimatic variables (see Table 1). For precipitation and total cloud cover, we suggest to carefully assess Fig. 4 if the region of interest is well represented by the statistics-based reconstruction. The actual numbers for the goodness of the fit and the model, *R*^2^ and *RMSE*, can be found in the diagnostic files listed in Table 5.

**Table 5:** List of diagnostic regression results that can be found in the *Open Science Framework* repository^28^ under the project’s data/coeffs directory.

hadcm3_000-120_jan_regression_temp_co2-ecospre-esinpre-obl.nc
hadcm3_000-120_feb_regression_temp_co2-ecospre-esinpre-obl.nc
…
hadcm3_000-120_dec_regression_temp_co2-ecospre-esinpre-obl.nc
hadcm3_000-120_ann_regression_temp_co2-ecospre-esinpre-obl.nc
hadcm3_000-120_jan_regression_prec_co2-ecospre-esinpre-obl.nc
hadcm3_000-120_feb_regression_prec_co2-ecospre-esinpre-obl.nc
…
hadcm3_000-120_dec_regression_prec_co2-ecospre-esinpre-obl.nc
hadcm3_000-120_ann_regression_prec_co2-ecospre-esinpre-obl.nc
hadcm3_000-120_jan_regression_tcc_co2-ecospre-esinpre-obl.nc
hadcm3_000-120_feb_regression_tcc_co2-ecospre-esinpre-obl.nc
…
hadcm3_000-120_dec_regression_tcc_co2-ecospre-esinpre-obl.nc
hadcm3_000-120_ann_regression_tcc_co2-ecospre-esinpre-obl.nc

These files contain useful statistical summary information such as *R*^2^, standard errors, or residuals for the individual, pixel-based linear regression models.

The quality of the underlying climate model, HadCM3, can be assessed in two ways. First, by looking at the model bias (Fig. 10), and second, by looking how well HadCM3 compares to proxy records that go well back in time and show the longer-term climatic changes happening on glacial–interglacial time scales (Figs. 6, 7, 8, 9). However, most geological proxies are useful for quantitative temperature comparisons, and only few, terrestrial proxies exist for precipitation, and they mostly reflect qualitative changes, e.g., wetter vs. drier periods. For cloud cover no such geological proxies exist.

## Usage Notes

Examples on how to access the NetCDF files of the reconstructed climate using *Python* or *R* are provided in the examples directory of the project repository^28^.

## Data Availability

Model code for the linear regression as well as the code for the analysis and visualisation of figures is publicly available in the project repository^28^. NetCDF files have been processed using cdo^30^. We used the Python language for most of our scripts with a few bash scripts as wrappers. The workflow for the data generation process is managed by *Snakemake*^31^. The linear regression is based on the statsmodels package^32^. All visualisations are made with matplotlib^33^ using cartopy^34^ for maps. Other Python packages used are (in alphabetical order): adjustText^35^, BeautifulSoup4 (https://www.crummy.com/software/BeautifulSoup/), netCDF4^36^, numpy^37^, pandas^38,39^, scipy^40^, scikit-image^41^, and tqdm^42^.
